# Complete Genome Sequence of Canine Papillomavirus Virus Type 12

**DOI:** 10.1128/genomeA.00294-15

**Published:** 2015-04-16

**Authors:** Dan Zhou, Jennifer Luff, Siddhartha Paul, Faris Alkhilaiwi, Yukari Usuda, Naidong Wang, Verena Affolter, Peter Moore, Richard Schlegel, Hang Yuan

**Affiliations:** a Department of Pathology, Georgetown University Medical School, Washington, DC, USA; b Department of Population Health and Pathobiology, College of Veterinary Medicine, North Carolina State University, Raleigh, North Carolina, USA; c Department of Pathology, Microbiology and Immunology, School of Veterinary Medicine, University of California Davis, Davis, California, USA

## Abstract

Papillomaviruses, of the family *Papillomaviridae*, are epitheliotropic, nonenveloped, circular, double-stranded DNA viruses that contribute to benign and malignant tumors in humans and animals. We report here the whole-genome sequence of canine papillomavirus type 12, found at a pigmented plaque located on the skin of a mixed-breed bloodhound.

## GENOME ANNOUNCEMENT

Papillomaviruses are nonenveloped double-stranded DNA viruses that are associated with both benign and malignant tumors in humans and animals. Human papillomaviruses (HPVs) cause cancers in the cervix, vulva, vagina, penis, anus, and oropharynx. In total, HPV infections caused 5.2% of all cancers worldwide in 2002 (1).

Papillomavirus (PV) infections are widespread in nature and occur in most mammalian species, as well as in birds and turtles (2). These viral infections are highly host and tissue tropic and are rarely transmitted between species. The existence of relevant mammalian models for PV infections has made possible studies of the evolution, life cycle, and pathogenesis of PVs. The canine model is one of the best systems for studying both epidermal and mucosal PV infections, and the analysis of canine oral papillomavirus type 1 (CPV-1) has been critical to understanding host immunity against papillomavirus infection (3, 4) and viral oncogenesis (5–8). More importantly, the CPV model has contributed significantly to development of the HPV vaccine (9, 10). To date, there are 11 canine PVs whose genomes have been sequenced (11), some of which are associated with subclinical infections that can clinically manifest during immunosuppression. PV infections in immunosuppressed dogs have also been noted to progress to aggressive squamous cell carcinomas.

Here, we report the complete genome sequencing of a novel type of canine papillomavirus, designated CPV-12, which was isolated from a solitary pigmented plaque on a mixed-breed bloodhound. The viral DNA was isolated from this tumor using routine methods. Initially, PCR with general primers was used to amplify potential PV genome fragments. The sequencing of the products revealed an unknown CPV type. To further pursue the type of CPV present, we used rolling circle amplification to generate the complete viral genome. The ampliﬁed viral genome was digested with BamHI, cloned into the BamHI site of the vector pUC19, and sequenced using primer walking-enabled sequencing of the entire viral genome from both directions. The analysis of the viral sequence was performed using ABI 3730xl DNA-analyzing instruments (Applied Biosystems) for capillary electrophoresis and ﬂuorescent dye terminator detection. The Vector NTI Advance 10 software (Invitrogen, USA) was used to assemble the sequence contigs containing high-quality trace ﬁles.

The complete genome sequence of CPV-12 is 7,890 bp. Similar to other papillomaviruses, CPV-12 has all of its open reading frames (ORFs) on the same coding strand of its circular double-stranded DNA genome. CPV-12 has seven ORFs that encode five early (E) proteins, E1, E2, E4, E6, and E7. In addition, there are two late (L) proteins, L1 and L2. The L1 gene is the most conserved gene within the papillomavirus genome and has therefore been used for the identification of new PV types. A new PV isolate is recognized if the DNA sequence of the L1 ORF differs by >10% from the closest known PV type (2). The L1 DNA of CPV-12 is most closely related (81% homology) to CPV-9. These data will facilitate future investigations of the evolutionary characteristics and molecular pathogenesis of CPVs.

### Nucleotide sequence accession number. 

The complete genome sequence of canine papillomavirus 12 (CPV-12) is available in GenBank under the accession no. JQ754321.
